# Rare, rarer, lung involvement in adult-onset Still's disease: A mini-review

**DOI:** 10.3389/fmed.2022.989777

**Published:** 2022-09-16

**Authors:** Jasper F. Nies, Udo Schneider, Martin Krusche

**Affiliations:** ^1^Internal Medicine II, University Medical Center Cologne, Cologne, Germany; ^2^Department of Rheumatology and Clinical Immunology, Charité - Universitätsmedizin Berlin, Berlin, Germany; ^3^Department of Nephrology, Rheumatology and Endocrinology, University Medical Center Hamburg-Eppendorf, Hamburg, Germany

**Keywords:** adult-onset Still's disease (AOSD), interstitial lung disease, pulmonary arterial hypertension, IL-6 blocking, IL-1 blocking

## Abstract

Adult-onset Still's disease (AOSD) is a polygenic systemic autoinflammatory disease which is associated with increased morbidity and mortality. Pulmonary involvement is a rare, but serious complication of AOSD. As in AOSD, IL-1b, IL-18, and IL-6 dominate the molecular pathogenesis, which mediate a type 1 and type 3 inflammatory signature of the adaptive immune system. This is evidenced by the success of IL-1- and IL-6 inhibition in the management of AOSD. However, anaphylactic reactions to treatment with IL-1- or IL-6-inhibitors is currently being discussed as a potential trigger for lung involvement inf AOSD, while genetic risk factors have also been identified. Clinically, pulmonary involvement in AOSD can manifest in many different forms. Parenchymal inflammation with peripheral consolidations is the most frequent form while PAH is less common, but often very difficult to manage. This mini-review provides an overview of the pathophysiology as well as the clinical presentation and the diagnostic features of pulmonary involvement in AOSD.

## Introduction

AOSD is a systemic autoinflammatory condition that manifests after the age of 16 years. Clinical hallmark features of this disease are high spiking fever, polyarthritis, and an evanescent, salmon-colored macular or maculopapular rash on the trunk and extremities during the fevers. However, involvement of almost every organ system has been reported.

Three major patterns, each affecting roughly one third of the patients, can be distinguished: (I) A monophasic pattern with systemic involvement and complete remission, (II) a recurrent pattern with systemic, but preferentially joint manifestations and a remitting / relapsing course, and (III) a chronic pattern that usually mainly affects the joints.

Mostly, the disease takes a mild course, but life-threatening complications occur in more than 20 % of the patients. Macrophage activation syndrome (MAS) is the most common serious complication. As an expression of a cytokine storm and overactivation of macrophages, it affects 10 to 19 % of AOSD patients and is characterized by fever, pancytopenia, coagulopathy, multiorgan involvement, and is associated with a mortality of 10–20 %.

Pulmonary involvement is a rarer, but serious complication of AOSD. As AOSD shares many characteristics with systemic juvenile idiopathic arthritis (sJIA)—the two are even seen as a continuum of the same disease (1, 2)—, it is noteworthy that lung involvement in sJIA has recently been suggested to be a distinct clinical entity called sJIA-lung disease (sJIA-LD) (3). As the awareness for sJIA-LD rises, it is worthwhile also investigating its adult counterpart, which might be referred to as AOSD-LD. This mini- review summarizes the most recent literature in order to shed some more light on this development.

## Epidemiology

Since AOSD is a rare disease, data on epidemiology is sparse. The incidence is estimated at 0.16 and 0.4 per 100.000 inhabitants (4, 5). There seems to be no gender preference and two age peaks have been identified: 15–25 years, and 36–45 years (5). However, the onset of the disease has also been reported in elder patients (6).

Lung involvement is seen in 5–12 % of AOSD patients (7, 8). While those with pulmonary manifestations are older than those without, risk factors for developing those manifestations in the first place include young age at diagnosis, profound lymphadenopathy and myalgia, strongly elevated leukocyte counts and levels of IL-18 and ferritin, previous episodes of MAS, and frequent recurrences of the disease (7–9). Also, lung involvement is associated with a significantly poorer prognosis: Ruscitti et al. reported a mortality of 38.9 % in AOSD patients with pulmonary affection compared to 10.1 % in patients without (8).

Very similar risk factors for and rates of sJIA-LD have been reported (3, 10). Interestingly, anaphylactoid reactions to biological drugs like anti-IL-1 and anti-IL-6 monoclonal antibodies have also been identified as a risk factor for sJIA-LD (10, 11): Since the use of these treatment options dramatically increased over the past years, this connection is alarming considering that the incidence of sJIA-LD, which has a mortality of up to 68 % (12), rose in parallel (10).

## Pathophysiology

As recently discussed (13), it is essential to understand the molecular mechanisms of immunological diseases to make tailored therapies possible. In this sense, AOSD seems to be mainly driven by an IL-1β-, IL-18-, and IL-6-mediated immune response, evidenced by great successes of IL-1- and IL-6-blockade (14, 15).

The molecular pathophysiology of AOSD is not completely understood yet, which of course includes the mechanisms of its pulmonary manifestations. However, despite the lack of good animal models, significant progress in understanding the disease has been made and reviewed in more detail elsewhere (16).

The activation of pattern-recognition receptors (PRR) seems to play a major role at the start of the process. Both pathogen-associated molecular patterns (PAMPs) from infectious agents and/or sterile danger-associated molecular patterns (DAMPs) are suspected to start the immunological reaction of AOSD by binding to PRRs, while the accumulation of the latter may be one explanation for the proposed link of malignancies to the development of the disease (17). The activation of the NOD-, LRR- and pyrin domain-containing protein 3 (NRLP3) inflammasome as an intracellular PRR and the following proteolytic activation of IL-1β and IL-18 stand at the center of AOSD pathophysiology (18–22). On the one hand, these cytokines induce further cytokines like IL-6, which leads to many of the characteristic clinical findings of AOSD. On the other hand, IL-18 and IL-1β induce two signaling cascades that integrate innate and adaptive immune responses with a predominance of macrophages and neutrophils (16). This is a consequence of a T-cell activation that most likely results from bystander activation in the context of a cytokine storm, facilitated by vulnerability due to certain HLA polymorphisms.

1. T_H_1 response: IL-18 activates T_H_1 cells leading to overproduction of IFNγ (18, 23) and induction of the chemokines CXCL9, CXCL10, and CXCL11 (24). This process is reinforced by an IL-18-mediated pathological secretion of IFNγ by natural killer (NK) cells, whose cytotoxic capacity is reduced in AOSD patients (25). This eventually leads to the potent activation of macrophages that is typical of AOSD and can spin out of control in the form of MAS.
2. T_H_17 response: IL-1β and IL-6 form a local cytokine milieu that skews T cell polarization in favor of T_H_17 cells (26), which is evidenced by reduced number of T_reg_ cells in the patients and results in a reduced capacity to control the inflammation (27). Signaling through Toll-like receptor 7 (TLR7) on dendritic cells supports this process (28). T_H_17 cells are potent pro-inflammatory cells and mediate tissue damage by recruiting neutrophils. CXCL8, a chemokine that attracts neutrophils and is induced by T_H_17 cells, is involved in the pathogenesis of AOSD (18). NETosis of neutrophils creates new DAMPs for PRRs, looping back to the beginning of the inflammatory process.

The result of this severe immunological reaction at the interface of the innate and adaptive immune systems is a cytokine storm with a potentially lethal consequence.

Concerning lung involvement of AOSD, macrophages, neutrophils, and CD4^+^ T cells have been identified in broncho-alveolar lavage (BAL) fluid and biopsy specimens (7, 29), underlining the similarity to the general pathophysiological concept of AOSD. However, the often-found predominance of neutrophils (7) is not a specific finding and should raise concern for more common mimickers like bacterial infections. mRNA expression analyses showed higher levels of *CXCL9* and *CXCL10* transcripts in patients with sJIA-LD (30). These chemokines are typically induced by IFNγ in macrophages and can recruit T_H_1 cells by binding to CXCR3. However, this finding has not be replicated for AOSD-LD as of yet. Still, protein levels of IL-1β, IL-18, IL-6, and ferritin are elevated in the blood of AOSD patients with lung involvement. Interestingly, Ruscitti et al. suggest that lung involvement might be the cause—instead of a collateral damage—of MAS, considering that the lung is a significant source of IL-1 and IL-6 (8).

Over the past years, a pathophysiological model for the development of pulmonary alveolar proteinosis (PAP) in sJIA-LD has formed. While the direct transferability to AOSD-LD is questionable as this pulmonary manifestation seems to be limited to the juvenile form, it is worthwhile mentioning that the massive immunological reaction of AOSD causes not only direct damage to the pulmonary tissue, but also affects alveolar macrophages in their ability to recycle surfactant. Additionally, hyperplasia of surfactant-producing type-II-pneumocytes has been reported in both AOSD-LD (31) and sJIA-LD (3). Even though no dysfunction in the GM-CSF homeostasis is seen (as would be typical of familial or sporadic PAP), this disequilibrium of production and cellular re-uptake leads to the accumulation of proteins in the alveolar space and the histopathological picture of PAP. There even is also some experimental evidence for this as T cell-restricted overexpression of *T-bet* (and subsequent increased production of IFNγ) leads to spontaneous PAP in mice (32).

Concerning the connection between drug reactions with eosinophilia and systemic symptoms (DRESS) to treatment with IL-1- or IL-6-inhibitor and the development of pulmonary manifestations, Saper et al. found a significant enrichment for HLA-DRB1^*^15 in a cohort of patients with Still's disease (both sJIA and AOSD) with lung involvement (33). To this end, T_H_2 activity and eosinophilia have also been described to be a pathophysiological puzzle piece.

**Figure 1:**
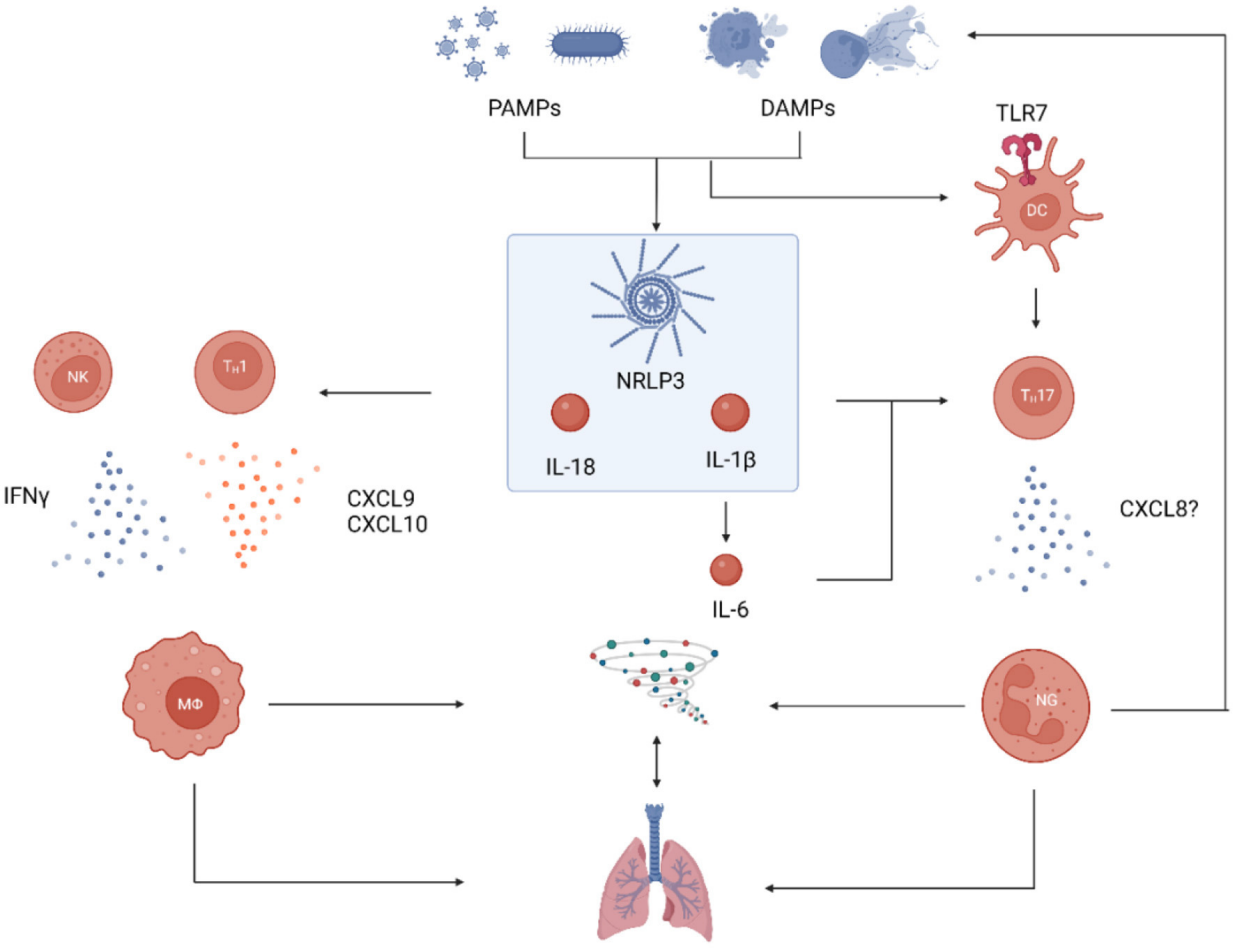
Proposed pathophysiological model for AOSD with lung involvement. Processes of general AOSD pathophysiology are shown in blue, those that have specifically been shown in pulmonary involvement are shown in red. PAMPs and DAMPs induce the immune reaction by binding to PRRs: Intracellular activation of the NRLP3 inflammasome and subsequent proteolytic activation of pro-IL18 and pro-IL-1β is a hallmark of the disease. IL-18 activates T_H_1 cells and stimulates NK cells to release IFNγ which in turn activates macrophages. These can recruit additional T_H_1 cells by secretion of the chemokines CXCL9 and CXCL10. IL-1β induces the production of IL-6 and the combination of the two skews the local cytokine milieu in favor of T_H_17 (over T_reg_) polarization. This process is supported by TLR7-signaling in DCs. T_H_17 cells recruit neutrophils which induce a potent inflammatory reaction that create even more DAMPs to reinforce the immunological cascade. This strong activation of both macrophages and neutrophils leads to a cytokine storm that can affect many organs, including the lungs. PAMP, pathogen-associated molecular pattern; DAMP, danger-associated molecular pattern; PRR, pattern recognition receptor; DC, dendritic cell; TLR7, toll-like receptor 7; NK, natural killer cell; MΦ, macrophage; NG, neutrophilic granulocyte (image created with BioRender, many thanks to Prof. Tobias B. Huber).

A pathophysiological hypothesis for AOSD and AOSD-LD is proposed in Figure 1.

Still, many questions remain. Links to other environmental factors like viral or bacterial infections (21) have also been suggested and seem likely given the activation of PRRs. However, they lack solid evidence, which includes the infection with (34) and the vaccination against SARS-CoV-2 (35). Hints to a genetic component from twin studies are weak (36, 37) and efforts to identify single nuclear polymorphisms or other common genetic changes by whole exome sequencing of several sJIA-LD patients (3) were futile. Of note, no genetic similarities to patients with PAP or MAS were found. However, several HLA alleles (38, 39) and gene polymorphisms (40) are associated with greater susceptibility for AOSD. The investigation of these aspects is limited by the lack of animal models and the retrospective character of most studies.

## Clinical presentation

Pulmonary manifestations of AOSD can be severe and even lethal. Conversely, the initial symptoms are often mild as patients mostly only report slight dyspnea (8), which is also true for sJIA-LD (3). This discrepancy makes it hard to detect AOSD-LD in its early stages to prevent severe manifestations that can follow. Thus, the existence of typical AOSD symptoms (high spiking fever, arthralgia, characteristic rash) in patients with even mild respiratory impairments should alarm the attending clinician.

Pulmonary symptoms include productive or dry cough, shortness of breath, pleuritic pain as a consequence of serositis, and cyanosis. Individual cases of alveolar hemorrhage with hemoptysis and aseptic empyema have been described (41, 42). Some patients with AOSD-LD report a sore throat 2 weeks before the onset of the pulmonary symptoms (31, 41). As a somewhat specific presentation of AOSD-LD, many reports mention that patients develop clubbed finger tips with digital erythema (3, 10).

Forty percent of the patients with lung involvement develop acute respiratory distress syndrome (ARDS), which is an early complication as 83 % of those develop the complication at or within the first year after initial diagnosis (7).

## Diagnostics

A typical aspect of AOSD-LD is a marked incongruence of fairly mild symptoms and severely deteriorated findings in laboratory parameters, functional tests, and pulmonary imaging. In terms of laboratory tests, patients with AOSD-LD typically show signs of autoinflammation which include strongly elevated ferritin with glycosylated ferritin levels of <20 %, elevated IL-6, IL-18, CRP, ESR, and liver enzyme levels (18). Anemia, leukocytosis with neutrophilia, lymphopenia, and eosinophilia are also common findings. While Takakuwa et al. saw no association with ferritin levels and pulmonary manifestation of AOSD (9), others reported significantly higher systemic clinical scores, more leukocytosis, and higher levels of ferritin in patients with affection of the lung (7, 8). Using serum proteome analyses of 162 patients, Chen et al. recently underlined the importance of IL-18 as it was stronger upregulated in sJIA patients with LD as compared to those without. Also, they identified ICAM5, a cell adhesion molecule, as a potential biomarker that is specifically upregulated in sJIA-LD (and not in general sJIA or MAS) and might be able to detect patients at risk at an early timepoint (43). However, this finding has not been replicated for AOSD-LD so far.

**Figure 2:**
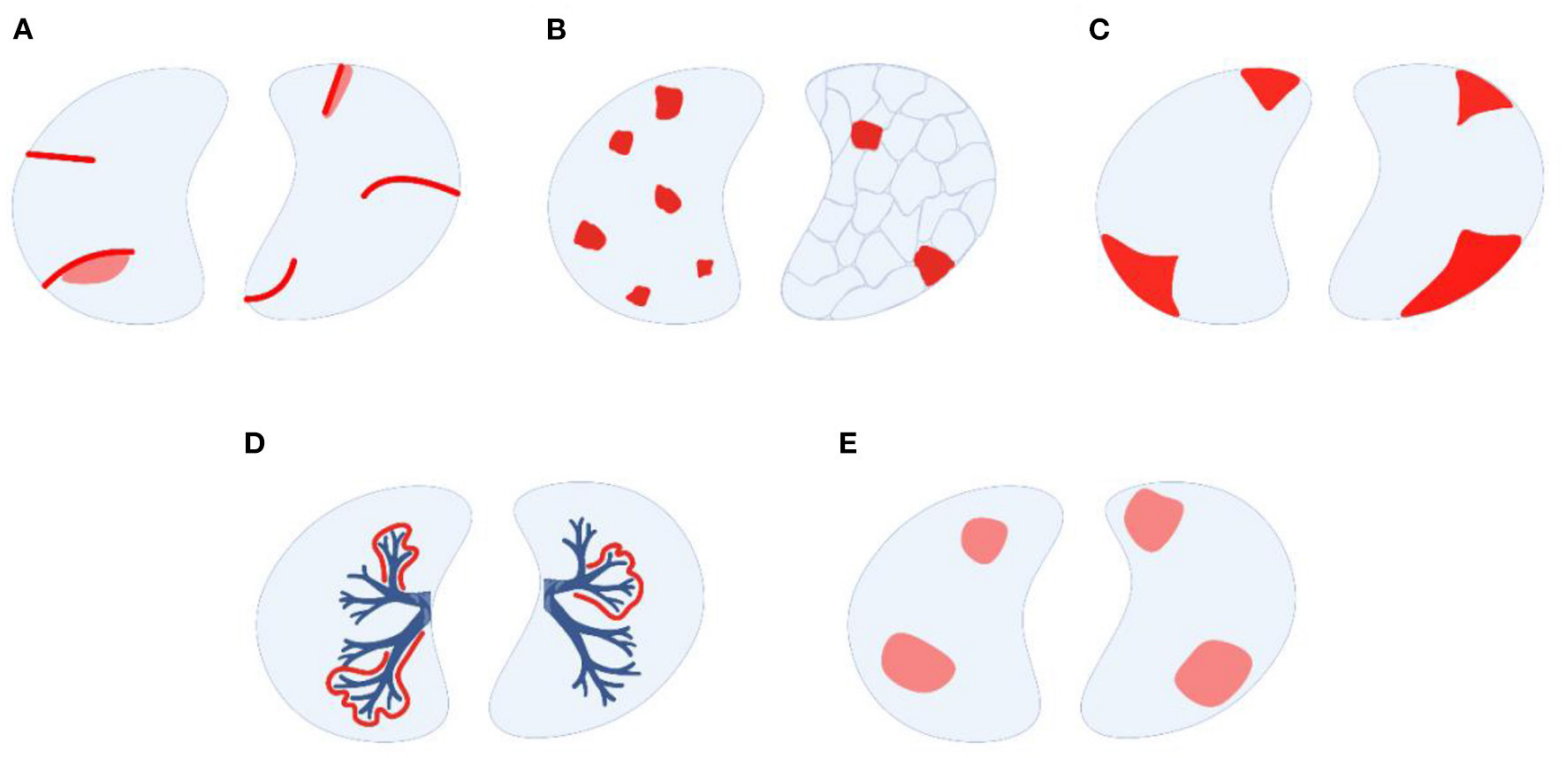
Modes of parenchymal lung involvement in AOSD-LD, modified from Rolfes and Kallinich (45). **(A)** peripheral septal thickening with or without adjacent ground-glass opacities, **(B)** crazy-paving pattern, **(C)** peripheral consolidations, **(D)** peribronchovascular consolidations, **(E)** predominantly ground-glass opacities (image created with BioRender, many thanks to Prof. Tobias B. Huber).

Considering both sJIA-LD and AOSD-LD, there are three main types of pulmonary manifestations: (I) airway / parenchymal, (II) vascular, and (III) alveolar.

(I) HR-CT pulmonary scans are the most important imaging modality to further categorize parenchymal pulmonary involvement in AOSD into five patterns (Figure 2), which are non-exclusive meaning that more than one may be present in a given patient (8): (A) multilobar, predominantly peripheral septal thickening with or without adjacent ground-glass opacities, (B) crazy paving pattern, (C) peripheral consolidations, (D) peribronchovascular consolidations, and (E) predominantly ground-glass opacities. Interestingly, pattern (C) is the most and (A) the least common presentation in AOSD-LD (8) while (A) is the most common one in sJIA-LD (10).

It is noteworthy that in contrast to many other ILDs attributed to autoimmune diseases AOSD-LD shows an exclusively inflammatory phenotype, which in general offers therapeutical options and a better prognosis than progressive fibrosing phenotypes: nevertheless, the prognosis is considerably poor in AOSD-LD.

Restrictive ventilation disorders and impaired diffusion capacity have been reported in several patients (42, 44, 45), which could be an expression of pathological changes to the parenchymal scaffold.

(II) Pulmonary arterial hypertension (PAH) is a less common, but can be severe (46) and hard to manage. In a Spanish AOSD cohort, its prevalence was 4.8 % with a high mortality of 22 % (47) while a recent case series with 13 patients even reports a mortality of almost 40 % (48). Cardiomegaly and dilation of atrium and chamber of the right heart hint at PAH in thoracic imaging. To further objectify these findings, echocardiography and right heart catheterization should be performed, which often uncover advanced pathological findings at diagnosis.
(III) Of note, BAL and biopsy studies of patients with sJIA-LD show striking similarities to PAP and endogenous lipoid pneumonia (ELP) with alveoli being filled with a substance rich in lipoproteins, cell detritus, cholesterol, and foamy macrophages, most likely as a consequence of the aforementioned imbalance in surfactant homeostasis. In imaging, crazy-paving pattern is a typical sign of PAP. As of yet, these typical, but non-specific, findings reported in the juvenile form have not been replicated in AOSD.

Mediastinal lymphadenopathy and exudative pleural effusion are further possible manifestations. Perry (49) and Qi (29) reported small centrilobular pulmonary nodules which entails further diagnostic workup to exclude neoplasia, usually by tissue sampling and histological examination.

Microscopic findings can yield a lymphoplasmacytic, non-granulomatous immune reaction as well as signs of intraalveolar (acute fibrinous) organizing pneumonia, bronchiolitis, and fibrotic remodeling (7, 49–51).

## Treatment

Due to its rarity and novelty, there are no evidence-based recommendations for the treatment of AOSD-LD. Thus, rational considerations and case-based reports are the current basis for therapy. Usually, prednisolone (0.5–1.0 mg/kg) is the first drug used to induce remission. Successful glucocorticoid-sparing agents in general AOSD include MTX and calcineurin-inhibitors (49, 52).

Alveolar hemorrhage (41) and PAH, however, tend to be refractory to glucocorticoid treatment. Intravenous immunoglobulins achieve some relief in refractory cases (31). In addition to general strategies with vasodilators, treatment with anakinra, ciclosporin A, or rituximab achieved clinical improvement in PAH (53–55).

As 20–30 % of AOSD patients are refractory to conventional therapies, biologicals are second-line options that should be considered (56). While TNFα blockade has turned out not to be very effective but shows effects on articular manifestations (57), IL-1 blockade is successfully used for management of systemic courses (58–60) and IL-6 blockade shows good efficacy for both joint involvement and systemic courses (61, 62). Recently, Zhang et al. reported an excellent response of a patient treated with tocilizumab as a rescue approach (14).

However, the treatment with biologicals might come at a risk. As mentioned above, the incidence of sJIA-LD, a condition very similar (if not the same) as AOSD-LD, increased in parallel to the increased clinical use of biologicals and anaphylactoid reactions to these drugs represent a risk factor for the development of sJIA-LD (9, 11). Since HLA-DRB1^*^15 was associated with an increased risk for such an allergic reaction (33), one might demand genetic testing in advance to the use of tocilizumab or anakinra in AOSD-LD. However, further studies are surely needed to clarify this connection.

## Outlook

AOSD-LD is an up to now poorly understood condition. Established registries for AOSD such as the Italian GIRRCS cohort (8) and the international AIDA registry (63) are a very valuable resource to investigate pulmonary manifestations from an epidemiological, clinical, and diagnostic standpoint. The development of animal models would be a great advancement to better characterize the pathophysiology of the disease and to test new therapeutics, which would be especially needed if the suspected triggering effect of anti-IL1- and anti-IL-6 treatment should turn out to be true. Luckily, new therapies to target central pathophysiological components of AOSD are already on the way: Emapalumab (64), an anti-IFNγ antibody, janus kinase (JAK) inhibitors (65–67), and tadekinig alfa (68), a recombinant IL-18 binding protein, are being evaluated for the treatment of AOSD.

## Summary

AOSD is a disorder mainly characterized by fever, arthritis, and a typical rash, but can affect almost any organ. Mainly driven by inflammasome activation, IL-18, and IL-1β, the pathophysiology can be located at the interface of innate and adaptive immune responses that lead to a cytokine storm. However, many blank spots in this model remain to be filled in. In parallel to its juvenile counterpart sJIA, lung involvement of AOSD (AOSD-LD) is a rare manifestation of the already rare disease. For the clinical practice it is worthwhile remembering that pulmonary involvement exists in the first place and that there is a striking discrepancy between rather mild clinical symptoms and profound changes in imaging. Concerning laboratory parameters, strongly elevated levels of ferritin, IL-18 and leukocytes are not only typical of AOSD, but also correlate with lung involvement. AOSD-LD can manifest in many different forms while parenchymal inflammation with peripheral consolidations on HR-CT imaging is the most frequent form. PAH is less common, but often diagnosed at late timepoints and is difficult to manage. Alveolar hemorrhage and aseptic empyema remain anecdotal, while pulmonary alveolar proteinosis seems to be limited to sJIA-LD and has not been reported in AOSD. Therapeutically, corticosteroids are initially used to control the severe inflammation, while methotrexate and calcineurin inhibitors showed good results as steroid-sparing agents. Refractory cases have successfully treated with biologicals, namely tocilizumab, anakinra, and canakinumab, which underlines the pathophysiological idea of IL-6 and IL-1 as central players in the disease. Already existing registries are powerful tools to understand this rare disease and its pulmonary manifestations better. However, animal models and more prospective studies would be a great asset in this endeavor.

## Author contributions

JN and MK: review concept and design and manuscript drafting. JN, US, and MK manuscript revision and final review. All authors approved the final version.

## Conflict of interest

Authors MK and US have received speakers and consultant honoraria by Sobi, Novartis and Roche/Chugai. The remaining author declares that the research was conducted in the absence of any commercial or financial relationships that could be construed as a potential conflict of interest.

## Publisher's note

All claims expressed in this article are solely those of the authors and do not necessarily represent those of their affiliated organizations, or those of the publisher, the editors and the reviewers. Any product that may be evaluated in this article, or claim that may be made by its manufacturer, is not guaranteed or endorsed by the publisher.
